# TLR4 modulates inflammatory gene targets in the retina during *Bacillus cereus* endophthalmitis

**DOI:** 10.1186/s12886-018-0764-8

**Published:** 2018-04-16

**Authors:** Phillip S. Coburn, Frederick C. Miller, Austin L. LaGrow, Salai Madhumathi Parkunan, C. Blake Randall, Rachel L. Staats, Michelle C. Callegan

**Affiliations:** 1 Department of Ophthalmology, University of Oklahoma Health Sciences Center, DMEI PA-419, 608 Stanton L. Young Blvd, Oklahoma City, OK 73104 USA; 2 Department of Family and Preventive Medicine, University of Oklahoma Health Sciences Center, Oklahoma City, Oklahoma USA; 3 Department of Cell Biology, University of Oklahoma Health Sciences Center, Oklahoma City, Oklahoma USA; 4 Oklahoma Center for Neuroscience, University of Oklahoma Health Sciences Center, Oklahoma City, Oklahoma USA; 5 Department of Microbiology and Immunology, Dean McGee Eye Institute, University of Oklahoma Health Sciences Center, Oklahoma City, Oklahoma USA

**Keywords:** Bacterial endophthalmitis, Retinal gene expression, Toll-like receptor 4, Gram-positive intraocular infections

## Abstract

**Background:**

Endophthalmitis is a serious intraocular infection that frequently results in significant inflammation and vision loss. Because current therapeutics are often unsuccessful in mitigating damaging inflammation during endophthalmitis, more rational targets are needed. Toll-like receptors (TLRs) recognize specific motifs on invading pathogens and initiate the innate inflammatory response. We reported that TLR4 contributes to the robust inflammation which is a hallmark of *Bacillus cereus* endophthalmitis. To identify novel, targetable host inflammatory factors in this disease, we performed microarray analysis to detect TLR4-dependent changes to the retinal transcriptome during *B. cereus* endophthalmitis.

**Results:**

C57BL/6 J and TLR4^−/−^ mouse eyes were infected with *B. cereus* and retinas were harvested at 4 h postinfection, a time representing the earliest onset of neutrophil infiltration. Genes related to acute inflammation and inflammatory cell recruitment including CXCL1 (KC), CXCL2 (MIP2-α), CXCL10 (IP-10), CCL2 (MCP1), and CCL3 (MIP1-α)) were significantly upregulated 5-fold or greater in C57BL/6 J retinas. The immune modulator IL-6, intercellular adhesion molecule ICAM1, and the inhibitor of cytokine signal transduction SOCS3 were upregulated 25-, 11-, and 10-fold, respectively, in these retinas. LIF, which is crucial for photoreceptor cell survival, was increased 6-fold. PTGS2/COX-2, which converts arachidonic acid to prostaglandin endoperoxide H2, was upregulated 9-fold. PTX3, typically produced in response to TLR engagement, was induced 15-fold. None of the aforementioned genes were upregulated in TLR4^−/−^ retinas following *B. cereus* infection.

**Conclusions:**

Our results have identified a cohort of mediators driven by TLR4 that may be important in regulating pro-inflammatory and protective pathways in the retina in response to *B. cereus* intraocular infection. This supports the prospect that blocking the activation of TLR-based pathways might serve as alternative targets for Gram-positive and Gram-negative endophthalmitis therapies in general.

**Electronic supplementary material:**

The online version of this article (10.1186/s12886-018-0764-8) contains supplementary material, which is available to authorized users.

## Background

Endophthalmitis is a serious infection of the posterior segment of the eye which occurs from introduction of microbes following a surgical procedure (post-operative endophthalmitis [POE]), a traumatic penetrating injury (post-traumatic endophthalmitis [PTE]), or bloodstream spread from an infection of a distant site in the body (endogenous endophthalmitis [EE]) [1–3]. Bacterial endophthalmitis is considered a medical emergency and often results in poor visual outcomes [4]. Much of the intraocular damage in endophthalmitis is due, in part, to the host inflammatory response [5–9]. Immediate and aggressive intervention to stop the progression of the disease is critical to salvaging vision. There is currently no universal therapeutic regimen which prevents the significant inflammation and vision loss associated with severe forms of endophthalmitis.

The Gram-positive pathogen *Bacillus cereus* is a leading cause of PTE and EE. PTE infections due to *B. cereus* progress rapidly and result in a fulminant endophthalmitis characterized by severe intraocular inflammation, eye pain, and loss of visual acuity within hours [1–4]. Complete blindness can result in 1 or 2 days, and in nearly half of these infections, evisceration or enucleation is required to salvage healthy tissue in the orbit [10]. The severity and rapid progression of this infection has been recapitulated in a mouse model [1, 2, 6–9]. Infection of mouse eyes with as few as 100 colony-forming units (CFU) of *B. cereus* results in significant inflammation and loss of visual function within hours, similar to that observed in human infections. Because inflammation in the eye causes damage to non-regenerative neural structures, it is important to identify host factors that lead to the events that contribute to this bystander damage.

Robust inflammation in response to intraocular bacterial infection is triggered by the early recognition of cellular components via a class of pattern recognition receptors called Toll-like receptors (TLRs) that are expressed on host cells [11, 12]. Parkunan et al. recently published findings implicating the TLR4/TRIF/MYD88 axis in intraocular *B. cereus* infections [8]. *B. cereus* infected eyes of TLR4^−/−^ mice had significantly less polymorphonuclear leukocytes (PMN) influx and reduced concentrations of four inflammatory mediators relative to infected eyes of C57BL/6 J wild type mice. These parameters correlated with a significant retention of retinal function. These results suggested that the inflammatory cascade in *B. cereus* endophthalmitis is initiated, in part, by TLR4 signaling through a potentially novel TLR4 ligand either expressed or induced by *B. cereus* [8].

The attenuated course of infection observed in TLR4^−/−^ mice implicated downstream mediators of the TLR4 pathway as important in the robust, early response in eyes infected with *B. cereus* [8]. In the current study, we sought to identify host TLR4-dependent factors upregulated in response to *B. cereus* intraocular infection. Based on previous observations of a less severe inflammatory response in TLR4^−/−^ mice [8], we hypothesized that the retinal gene expression profile would be significantly different between TLR4-deficient mice and C57BL/6 J mice following infection. Microarray analysis identified 15 genes involved in the acute inflammatory response, neutrophil recruitment, photoreceptor cell survival, and pathogen recognition and clearance that were upregulated 5-fold or greater in infected C57BL/6 J wild type mice compared to their levels in uninfected control mice (Table 1). The expression of 14 out of 15 of these genes was found to be unaltered in TLR4^−/−^ mice relative to uninfected controls, indicating their dependency on TLR4 (Table 1). These genes included key mediators in neutrophil recruitment, and activation of photoreceptor survival and pathogen clearance mechanisms in response to *B. cereus* infection. These results further suggest that the TLR4 pathway might serve as a target for new anti-inflammatory treatments critically needed to not only control the explosive inflammation seen in *B. cereus* ocular infection, but also ocular infections due to *Klebsiella pneumoniae* and other Gram negative pathogens.

**Table 1:** Microarray analysis of retinal genes upregulated 5-fold and higher 4 h postinfection with *B. cereus* ATCC14579

Gene symbol	Gene title	RefSeq Transcript ID	Fold-Change (C57BL6/J infected versus uninfected)	*p*-value	Fold-Change (TLR4^−/−^ infected versus uninfected)	*p*-value
CXCL1	chemokine (C-X-C motif) ligand 1	NM_008176	34	0.0106	NC	NS
CXCL2	chemokine (C-X-C motif) ligand 2	NM_009140	29	0.0225	NC	NS
IL-6	interleukin 6	NM_031168	25	0.0114	NC	NS
CXCL10	chemokine (C-X-C motif) ligand 10	NM_021274	21	0.0328	NC	NS
CCL2	chemokine (C-C motif) ligand 2	NM_011333	20	0.0355	NC	NS
CCL3	chemokine (C-C motif) ligand 3	NM_011337	16	0.0006	5	0.0446
PTX3	pentraxin related gene	NM_008987	15	0.0376	NC	NS
ICAM1	intercellular adhesion molecule 1	NM_010493	11	0.0026	NC	NS
SOCS3	suppressor of cytokine signaling 3	NM_007707	10	0.0034	NC	NS
CYR61	cysteine rich protein 61	NM_010516	10	0.0116	NC	NS
MOBP	myelin-associated oligodendrocytic basic protein	NM_008614	NC	NS	10	0.0270
MBP	myelin basic protein	NM_001025245	NC	NS	10	0.0223
PTGS2	prostaglandin-endoperoxide synthase 2	NM_011198	9	0.0027	NC	NS
STEAP4	STEAP family member 4	NM_054098	6	0.0116	NC	NS
LIF	leukemia inhibitory factor	NM_008501	6	0.0055	NC	NS
CH25H	cholesterol 25-hydroxylase	NM_009890	6	0.0232	NC	NS
PLP1	proteolipid protein (myelin) 1	NM_011123	NC	NS	6	0.0236
EGR2	early growth response 2	NM_010118	5	0.0062	NC	NS

The fold changes of retinal genes in C57BL6/J mouse eyes and TLR4−/− mouse eyes after infection relative to the uninfected, contralateral eye are shown. Levels of significance were determined using ANOVA and *p* < 0.05 was considered significant

*NC* no change, *NS* not significant

## Methods

### Animals and ethics statement

*This study was carried out in strict accordance with the recommendations in the Guide for the Care and Use of Laboratory Animals of the National Institutes of Health. The protocol was approved by the Institutional Animal Care and Use Committee of the University of Oklahoma Health Sciences Center (protocol number 16–086).* Six week old C57BL/6 J (wild type) mice were acquired from the Jackson Laboratory (Catalog 000664, Bar Harbor ME) and age-matched, homozygous TLR4^−/−^ mice on the C57BL/6 J background were acquired from Eric Perlman, Case Western University, with the permission of S. Akira [13]. Mice were allowed to adjust to conventional housing 2 weeks prior to injection to equilibrate their microbiota. Mice were anesthetized with a cocktail of 85 mg ketamine/kg and 14 mg xylazine/kg prior to injections of bacteria. Mice were euthanized by CO2 inhalation.

### Experimental *B. cereus* endophthalmitis

Wildtype *B. cereus* ATCC 14579 was grown to early stationary phase in BHI broth for 18 h and diluted to 100 CFU/0.5 μl for injection into the mid-vitreous of right eyes. The left eyes served as uninjected controls [5–9].

### Microarray analysis of retinal gene expression

At 4 h postinfection, retinas were dissected from all eyes and were immediately frozen. Total RNAs were isolated from the frozen retinas using the Qiagen RNeasy Mini kit (Qiagen, Valenica, CA) following the manufacture’s instruction with on-column DNase treatment. RNA concentrations were measured using a Nanodrop ND-1000 Spectrophotometer and RNA quality was verified with an Agilent 2100 Bioanalyzer using an RNA Nano Chip. All RNA samples displaying no visible degradation in the Bioanalyzer analysis with two sharp ribosomal peaks were deemed acceptable for further processing. Affymetrix’s GeneChip IVT Express kit was used for cDNA synthesis and in vitro transcription. Affymetrix GeneChip Mouse Genome 430 2.0 Array was used in this study and the raw image was acquired by scanning the arrays using GeneChip scanner. Multiple files were generated and exported by Affymetrix’s software Command console. These files were used for subsequent Bioinformatics analysis. Data analysis was performed using Partek’s Genomics Suite software (Partek Inc., St. Louis, Missouri). A 5-fold change in gene expression and *p* < 0.05 threshold were selected as the criteria for comparative array analyses. Arrays were performed in duplicate on independently obtained RNA samples (SeqWright Genomic Services, Houston, TX).

### RNA preparation for quantitative real time PCR

At 4 h postinfection, infected and uninfected mice were euthanized and the retinal tissue harvested. Retinal tissue was placed in a 1.5 ml screw-cap tube containing 500 μl Sigma Tri reagent and 500 μl 1.0 mm glass beads (Biospec Products, Bartlesville, OK). After homogenization at 5000 rpm for 60 s, the supernatant was transferred to a sterile, nuclease free tube containing 200 μl chloroform and then mixed, incubated on ice, and centrifuged. The supernatant was added to 500 μl isopropyl alcohol and placed at -80 °C for 2 h to precipitate RNA. After centrifugation at 14,000 rpm for 20 min at 4 °C, the supernatant was discarded, the pellet washed with 500 μl 75% ethanol, and vortexed to resuspend the pellet. Following centrifugation at 12,000 rpm for 15 min at 4 °C, the supernatant was discarded and the pellet dried and then resuspended in 25 μl nuclease-free water. The concentration and purity were checked on a Nanodrop spectrophotometer and if necessary DNA contamination removed using the TURBO DNA free kit per the manufacturer’s instructions (Thermo Fisher Scientific, Waltham, MA).

### Quantitative real time PCR analysis

Total RNA was isolated and an aliquot of 50 ng of RNA was subjected to qPCR using the iTaq™ Universal SYBR^®^ Green One-Step kit (Bio-Rad, Hercules, CA), PrimeTime^®^ qPCR primers (Integrated DNA Technologies, Inc., Coralville, Iowa) specific to the mouse genes shown in Table 2 such that each primer was present in a final concentration of 300 nM, and a Bio-Rad^®^ CFX96 Touch™ Real-Time PCR System (Bio-Rad). Primer sequences are listed in Additional file 1: Table S2. Dissociation curves were used to assess the successful amplification of the desired product, and the threshold cycle (C_T_) was used to determine relative amounts of transcripts between RNA samples from infected and uninfected eyes. Each RNA sample was normalized using an internal actin gene control. Fold increases were calculated by subtracting the C_T_ values of the infected samples from the C_T_ values of the uninfected samples. That value as a power of 2 yielded the fold increase of the transcript from the infected sample relative to the uninfected sample. Eleven genes that were identified by microarray analysis as upregulated 5-fold or greater were chosen for validation. For each gene, qPCR was performed in triplicate on two independently prepared RNA samples. Reported fold increases of transcription represent the mean fold increase ± SD.

**Table 2:** Quantitative PCR confirmation of retinal gene expression 4 h postinfection with *B. cereus* ATCC14579

Gene symbol	C57BL/6 J Fold change	TLR4^−/−^ Fold change
CCL2	515 ± 742	NC
IL-6	118 ± 157	NC
CXCL2	56 ± 37	NC
CCL3	47 ± 31	NC
PTX3	32 ± 36	NC
CXCL1	30 ± 23	NC
LIF	29 ± 33	NC
CXCL10	27 ± 16	NC
ICAM1	16 ± 10	NC
SOCS3	12 ± 7	NC
PTGS2	6 ± 4	NC

C57BL/6 J fold change values are relative to uninfected C57BL/6 J mice eyes, and TLR4^−/−^ fold change values are relative to uninfected TLR4^−/−^ mice eyes. For each gene, qPCR was performed in triplicate on two independently prepared RNA samples. Significant differences between infected and uninfected eyes were assessed using a Paired T-test, and statistical significance was defined as *p* < 0.05

*NC* no change

### Statistics

Microarray analysis was performed using Partek’s Genomics Suite (Partek Inc., St. Louis, Missouri) software to obtain differential gene expression data. All data were normalized using the Robust Multi-array Analysis expression statistical analysis (RMA). Analysis of variance (ANOVA) was used to compare the means of the infected versus uninfected groups. A 5-fold change in gene expression and *p* < 0.05 threshold were selected as the criteria for comparative array analyses. For analysis of the qPCR data, ΔC_T_ values were calculated by subtracting the CT values of the infected samples from the uninfected samples. Significant differences between the mean ΔC_T_ values from infected and uninfected groups were assessed by a Paired T-test using GraphPad Prism 6.05 (GraphPad Software, Inc., La Jolla CA). Statistical significance was *p* < 0.05.

## Results

### Identification of TLR4-dependent genes upregulated in the retina following *B. cereus* intraocular infection

**Fig. 1:**
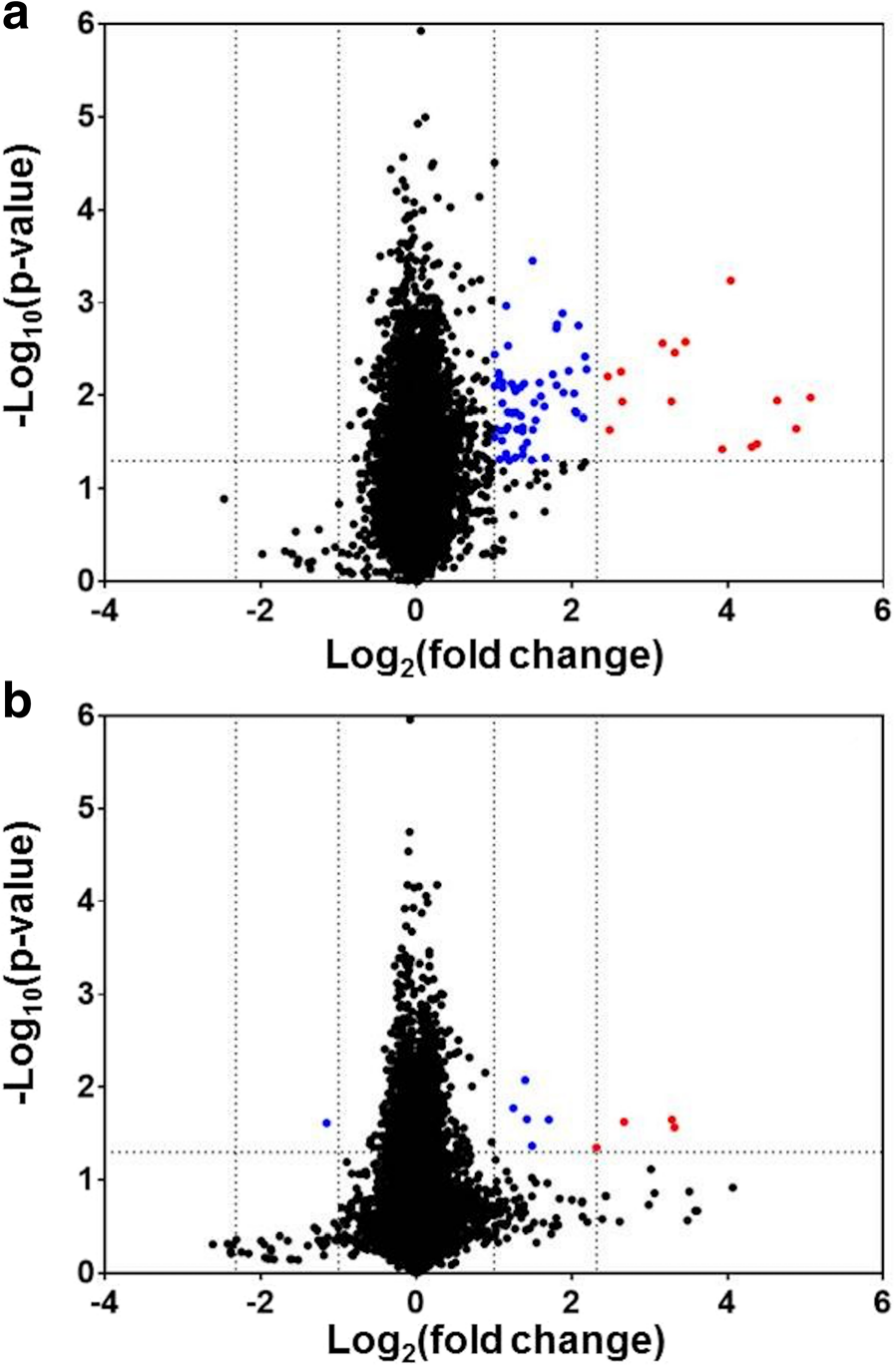
A volcano plot analysis of microarray data derived from C57BL/6 J (**a**) and TLR4^−/−^ (**b**) retinas 4 h postinfection with *B. cereus.* The x-axis indicates the log fold change and the y-axis indicates the negative log10 *p*-value. Each dot represents an individual gene, with blue dots depicting genes 2- to 4.9-fold upregulated, and red dots depicting genes 5-fold or greater upregulated in infected mouse eyes relative to uninfected eyes. Significance was assessed using ANOVA and a *p* value of < 0.05 was considered significant

**Fig. 2:**
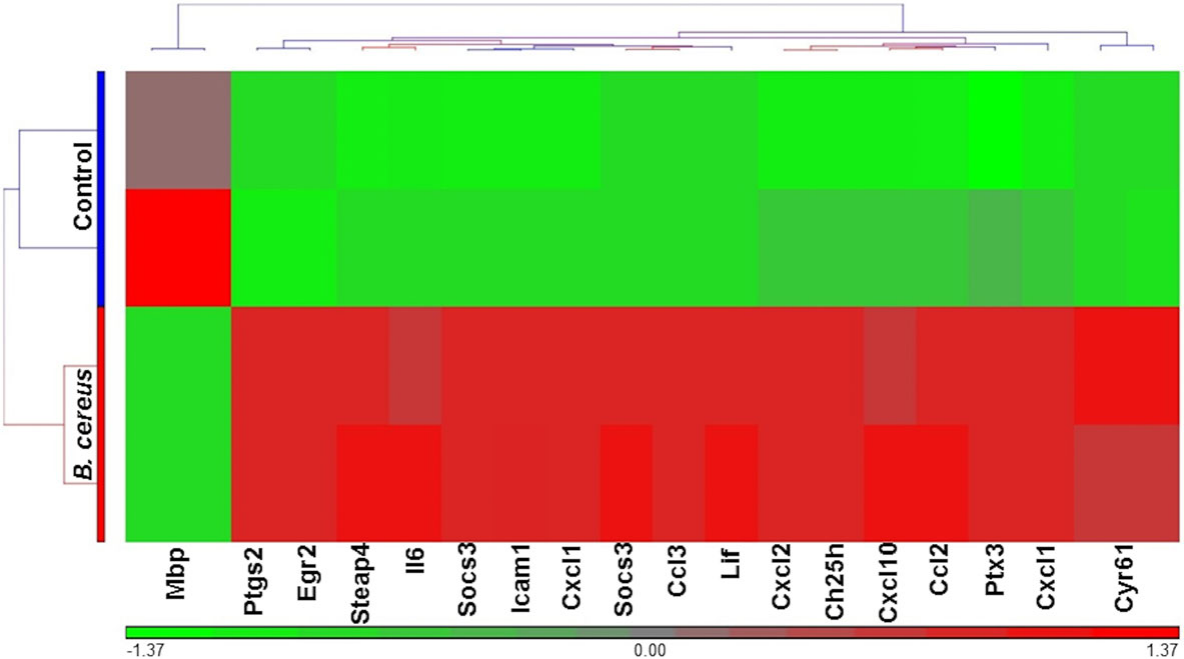
Hierarchical clustering dendogram of genes upregulated 5-fold or greater in control C57BL6/J, uninfected eyes and in C57BL6/J mouse eyes 4 h following infection with *B. cereus* indicating the relatedness of upregulated genes. The horizontal color bar at the bottom of the heat map indicates that different colors in the heap map represent gradients of gene expression levels: red, up-regulated expression; green, down-regulated expression; grey, no difference in gene expression. The branch lengths on the top of the heat map indicate the correlation with which genes were joined, with longer branches indicating a lower correlation

Because *B. cereus* infection of the posterior segment of the eye results in a rapid and vigorous inflammatory response, we sought to identify inflammatory mediators that are expressed early in the course of *B. cereus* infection. RNA was obtained from retinas at 4 h postinfection, which represents a time prior to neutrophil infiltration of the eye. We identified 76 genes whose expression was significantly altered 2-fold or greater in infected eyes relative to control eyes (Additional file 2: Table S1, Table 1, Fig. 1a). Figure 1a depicts a volcano plot analysis of the resultant microarray data (Fig. 1a). While a 2-fold change in expression represents a common statistical cutoff value, we focused on genes whose expression changed 5-fold or more to identify genes whose expression patterns changed the most dramatically early during infection. The clinical manifestations associated with *B. cereus* ocular infection progress rapidly, therefore genes upregulated to the highest degree early during infection might represent potential treatment targets. We identified 15 genes that were upregulated 5-fold or greater in C57BL6/J mice (Table 1, Figs. 1a and 2).

To ascertain whether the expression of these genes was dependent on TLR4, we performed the same experiments described above in TLR4^−/−^ mice. As shown in Fig. 1b and Table 1, none of the genes identified in wild type retinas at 4 h postinfection were significantly upregulated in TLR4^−/−^ retinas, with the exception of CCL3 (upregulated 5-fold). Conversely, we observed 3 genes that were significantly upregulated in the TLR4^−/−^ retinas but not upregulated in C57BL/6 J wild type mice. Myelin-associated oligodendrocytic basic protein (MOBP) and myelin basic protein (MBP) were upregulated 10-fold, and the proteolipid protein 1 (PLP1) was upregulated 6-fold at 4 h postinfection in infected TLR4^−/−^ eyes (Table 1).

### Upregulation of TLR4-dependent inflammatory chemokines and cytokines after *B. cereus* ocular infection

Key proinflammatory chemokines and cytokines were identified among the genes showing high level expression after *B. cereus* infection of wild type eyes. These included the inflammatory mediators chemokine (C-X-C motif) ligand 1(CXCL1) (keratinocyte chemoattractant [KC]), chemokine (C-X-C motif) ligand 2 (CXCL2) (macrophage inflammatory protein 2-alpha [MIP2-α]), chemokine (C-X-C motif) ligand 10(CXCL10) (interferon gamma-induced protein 10 [IP-10]), chemokine (C-C motif) ligand 2 (CCL2) (monocyte chemoattractant protein 1 [MCP1]), and chemokine (C-C motif) ligand 3 (CCL3) (macrophage inflammatory protein 1-alpha [MIP1-α]). These genes were all upregulated more than 16-fold by microarray analysis and are related to the acute proinflammatory response and inflammatory cell recruitment (see Additional file 3: Figure S1). The expression of CXCL1 was increased 34-fold, CXCL2 was increased 29-fold, and CXCL10 was increased 21-fold in C57BL/6 J mouse eyes when compared to uninfected control eyes. The expression of CCL2 was increased 20-fold and the expression of CCL3 was increased 16-fold. The expression of interleukin 6 (IL-6)**,** a powerful chemoattractant, was increased 25-fold when compared to uninfected control eyes. This group of potent, proinflammatory chemokines and cytokines were the most highly upregulated of all the significantly upregulated genes (Table 1).

Microarray analysis of the 4 h transcriptome in *B. cereus-*infected TLR4^−/−^ retinas demonstrated that only 1 out of 6 of these chemokines and cytokines, CCL3, was significantly upregulated. Quantitative PCR confirmed upregulation of CXCL1, CXCL2, CXCL10, CCL2, CCL3, and IL-6 in wild type C57BL6/J mouse retinas following infection relative to uninfected mouse eyes, but no changes in expression in any of these genes in TLR4^−/−^ mouse retinas 4 h after infection were observed, including CCL3 (Table 2, primer sequences are shown in Additional file 1: Table S2). There was a 30-fold change in the expression of CXCL1, a 56-fold change in CXCL2 expression, and a 27-fold change in the expression of CXCL10 when compared to uninfected control mice. Further, we demonstrated a 515-fold change in the expression of CCL2, a 47-fold change in CCL3, and a 118-fold change in IL-6 by quantitative PCR (Table 2). The only disagreement between the microarray and qPCR was in CCL3 expression.

### Upregulation of TLR4-dependent mediators of neutrophil recruitment and complement activation after *B. cereus* ocular infection

Inflammatory mediators involved in neutrophil recruitment and pathogen recognition and clearance were also significantly upregulated 5-fold or greater by microarray analysis. Intercellular adhesion molecule 1 (ICAM1), an adhesin expressed on endothelium and necessary for neutrophil diapedesis [14, 15], was upregulated 11-fold (Table 1 and Additional file 4: Figure S2). The extracellular matrix protein, cysteine rich protein 61 (CYR61), was increased 10-fold following infection. CYR61 also plays an important role in adhesion, chemotaxis and migration (Table 1 and Additional file 3: Figure S1) [16]. The expression of pentraxin 3 (PTX3), which has been shown to activate the classical complement pathway via C1q, and facilitate pathogen recognition and clearance [17, 18], was increased 15-fold (Table 1 and Additional file 3: Figure S1). Quantitative PCR confirmed upregulation of ICAM1 and PTX3 by 16- and 32-fold, respectively, after *B. cereus* infection of C57BL6/J mice (Table 2). Microarray analysis and qPCR did not detect significant changes in ICAM1, CYR61, or PTX3 transcript levels in TLR4^−/−^ following infection (Tables 1 and 2), suggesting the importance of TLR4 in eliciting a neutrophil response and complement activation following *B. cereus* ocular infection.

### Upregulation of TLR4-dependent inflammatory regulators after *B. cereus* ocular infection

Regulators of the inflammatory response were also found to be upregulated by greater than 5-fold following intraocular infection with *B. cereus.* The suppressor of cytokine signaling 3 (SOCS3) was upregulated 10-fold in wild type retinas following infection (Table 1 and Additional file 5: Figure S3). SOCS3 regulates signal transducer and activator of transcription 3 (STAT3) activation in response to cytokines [19]. Leukemia Inhibitory Factor (LIF), an IL-6 regulated neurocytokine was upregulated by 6-fold (Table 1 and Additional file 5: Figure S3). STEAP4 (six transmembrane epithelial antigen of prostate, family member 4), a metalloreductase that plays a role in inflammatory cytokine regulation [20], was increased by 6-fold (Table 1 and Additional file 4: Figure S2). The transcription factor early growth response 2 gene (EGR2) was upregulated 5-fold (Table 1 and Additional file 5: Figure S3). The cyclooxgenase isoenzyme (COX-2 or PTGS2) and cholesterol 25-hydroxylase (CH25H) were increased by 9- and 6-fold respectively (Table 1 and Additional file 3: Figure S1 and Additional file 5: Figure S3). Quantitative PCR confirmed the array results for SOCS3, LIF, and PTGS2 (Table 2). Significant changes in these genes were not observed following infection in TLR4^−/−^ mice (Tables 1 and 2).

## Discussion

*B. cereus* infection of the eye leads to a rapid and destructive inflammatory response that has devastating consequences for vision. TLR2 and TLR4 are key mediators of the innate immune response to bacterial pathogens during the early stages of endophthalmitis [21, 22]. TLR2 plays an important role in both *B. cereus* [21] and *S. aureus* [22] endophthalmitis. TLR4 also plays a significant role in mediating inflammation in *B. cereus* endophthalmitis [8] and, as expected, in *Klebsiella pneumoniae* endophthalmitis [23]. Parkunan et al. [8] demonstrated an increase in TLR4 mediated chemokines and inflammatory markers following *B. cereus* intraocular infection. Levels of the chemokines CXCL1 (KC), TNF-α (tumor necrosis factor alpha), IL-6, IL-1β were significantly reduced in TLR4^−/−^ mice when compared to wild type mice infected with *B. cereus* [8]. These findings corroborate our microarray and qPCR results. Our previous studies assessed the expression of a limited set of proinflammatory chemokines and cytokines and used whole globes for analysis, which did not permit identification of the source of proinflammatory mediators. Using a transcriptomics approach, we identified 15 proinflammatory and immunomodulatory TLR4-dependent genes whose expression was increased 5-fold or greater specifically in the retinas of eyes 4 h after infection with *B. cereus*. Since these genes were highly upregulated early during the course of infection, they potentially represent targetable host factors that mediate the initial response to *B. cereus* ocular infection.

In the current study, CXCL1 (KC) was the most highly upregulated gene by microarray analysis in the retinas of eyes infected with *B. cereus*. Quantitative PCR confirmed upregulation of CXCL1. Elevated CXCL1 expression was not surprising, considering its chemoattractant properties and the neutrophil burden observed in the posterior segment following infection [9]. We also observed upregulation of CXCL2 (MIP-2α), CXCL10 (IP-10), and CCL2 (MCP1) chemokines in these retinas. CXCL2 is highly homologous and shares many of the same roles in acute inflammation as CXCL1, including interaction with the CXCR2 receptor, secretion by monocytes and macrophages**,** and attraction of neutrophils to sites of infection and inflammation [24–26]. CXCL10 is secreted by monocytes, endothelium, and fibroblasts after IFN-γ (interferon gamma) stimulation in response to viral infection, and after LPS stimulation in response to Gram-negative infection. CXCL10 serves as a chemoattractant that recruits monocytes/macrophages, T cells, NK cells, and dendritic cells [27, 28]. Since CXCL10 production can occur as a result of stimulation by LPS through TLR4 [27], we hypothesize that the observed upregulation of CXCL10 after *B. cereus* infection may have resulted from the activation of TLR4 by novel ligand. Given that *B. cereus* is a Gram-positive bacterium and does not produce LPS, CXCL10 upregulation might occur due to the activation of TLR4 by another ligand produced or elicited by *B. cereus*. Rajamani et al. did not observe CXCL10 upregulation following intraocular infection with *S. aureus*, however inflammation in this infection is primarily driven by TLR2, and not by TLR4 [29].

IL-6 expression was upregulated 25-fold in C57BL6/J mice on the microarray analysis relative to uninfected control mice and was not upregulated in TLR4^−/−^ mice relative to uninfected controls. IL-6 is both a proinflammatory chemoattractant and a modulator of inflammation via signaling that increases the expression of TNF-α and IL-1β antagonists. Rajamani et al., demonstrated that IL-6 and IL-1β were both significantly upregulated during *S. aureus* endophthalmitis and suggested that these genes were important for the response to *S. aureus* infection [29]. Parkunan reported that IL-6 levels were markedly increased at 8 and 12 h postinfection in a TLR4-dependent manner following intraocular infection with *B. cereus* [8]. In contrast to the findings of Parkunan et al., we did not detect increased levels of TNFα transcript in retinas at 4 h following infection. This suggests that the source of TNFα seen by Parkunan may not be from cells in the retina at this stage of infection, but rather from infiltrating neutrophils, given that cytokine assays were performed on homogenized whole globes [8]. Neutrophils enter the eye as early as 4 h postinfection with *B. cereus*, but do not infiltrate the retinal layers until approximately 8 h postinfection [6]. IL-6 is a pro-inflammatory mediator expressed when bacterial recognition induces inflammation via TLR4 activation. While IL-6 has also been shown to be anti-inflammatory due to its ability to induce soluble TNFα and IL-1β receptor antagonist expression [30], this is unlikely the case in our model, given that neither TNFα or IL-1β expression was altered in the retina at 4 h. The increase in IL-6 seen in our microarray analysis done at 4 h implicates IL-6’s inflammatory role as a chemoattractant, while not precluding its role as a signal to downregulate TNF-α and IL-1β expression at a later time point as a means to limit inflammation thus preserving susceptible cells in the retina.

IL-6 is expressed by several cell types in the eye, including RPE cells, ganglion cells, and resident microglia [31, 32]. Others have reported that levels of IL-6 are markedly increased in the retina upon damage to the optic nerve [33], while others have reported IL-6 protects mature retinal ganglion cells from pressure-induced death [34]. In contrast, IL-6 was dispensible for an inflammatory response following *B. cereus* infection of the eye. A similar course of infection, proinflammatory mediator profile, neutrophil infiltration, and architectural changes to the retinal layers were observed in both IL-6^−/−^ and C57BL/6 J eyes [9]. Redundancy due to additional gp130-dependent cytokines, such as LIF [19], which is expressed at significantly higher levels in our analysis, might explain why the inflammatory response in IL-6^−/−^ mouse eyes was not significantly dampened following *B. cereus* infection. Additionally, the increased expression of IL-6 seen in this experiment might serve the dual role of helping to initiate the early inflammatory response seen in *B. cereus* endophthalmitis as well as serving to protect sensitive neuroretinal cells by limiting further inflammation and preventing apoptosis, as has been shown in glaucoma models [34].

CCL2 recruits monocytes, basophils, memory T-cells, and dendritic cells, but not neutrophils or eosinophils [35, 36], and CCL3 activates neutrophils [24]. CCL2 and CCL3 are critical to the recruitment of neutrophils in the context of keratitis [37]. In a mouse model of *Pseudomonas aeruginosa*-induced corneal infection, antibodies directed against CCL2 or CCL3 significantly reduced neutrophil infiltration into the cornea and decreased corneal damage. Both CCL2 and CCL3 were upregulated in the context of TLR4-mediated inflammation after *B. cereus* ocular infection, and their blockade might reduce neutrophil infiltration into the vitreous and decrease damage to the retina.

While the mechanisms of neutrophil infiltration into the eye during endophthalmitis are not completely understood, *S. aureus* is capable of inducing expression of E-selectin and ICAM1 on macrovascular endothelial cells in a rat model of endophthalmitis [38]. In the current study, we demonstrated that *B. cereus* infection also induces ICAM1 expression in the retina. Expression of ICAM1 is upregulated by a variety of stimuli including retinoic acid, oxidant stress, and the proinflammatory cytokines IL-1β, TNFα, and IFNγ [39]. Lipopolysaccharide (LPS) was shown to induce expression of ICAM1 in human pulmonary alveolar epithelial cells through the TLR4/c-Src/NADPH oxidase/ROS-dependent NF-κB pathway [40]. ICAM1 serves as the ligand for LFA-1 (Lymphocyte function-associated antigen 1integrin) on leukocytes [14, 15]. Leukocytes that bind to endothelium via ICAM1 / LFA-1 complex are able to initiate transmigration across the endothelial membrane [41]. The finding that ICAM1 is upregulated in wild type, but not in TLR4^−/−^ eyes, correlates with our previous findings that neutrophil recruitment is decreased in TLR4^−/−^ eyes following infection [8]. Interestingly, ICAM1 possesses signal-transducing functions that are associated primarily with proinflammatory pathways. Ligation of ICAM1 on the surface of endothelial cells elicits a signaling cascade resulting in production of IL-8 and RANTES (regulated on activation, normal T cell expressed and secreted) [42], as well as additional ICAM1 in a positive feedback loop [43]. Upregulation of ICAM1 early during infection and prior to PMN infiltration might indicate an additional role in signaling at this stage in addition to leukocyte trafficking.

Another gene product implicated in leukocyte migration, cysteine-rich protein 61 (CYR61), was also significantly upregulated following *B. cereus* infection. CYR61 is a modular protein that functions as a bridge between cells and the extracellular matrix, binding to integrins and to extracellular matrix proteins [16]. CYR61 is primarily involved in regulating adhesion and chemotaxis, as well as angiogenesis [16]. In contrast to the rapid and significant upregulation of CYR61 following *B. cereus* infection, Rajamani and colleagues did not observe upregulation of CYR61 until 12 h after intraocular infection with *S. aureus* [29]. This finding correlates with the delay in neutrophil influx observed in *S. aureus* endophthalmitis as compared to *B. cereus* endophthalmitis. It is likely that CYR61 is upregulated in order to mediate neutrophil invasion following *B. cereus* infection of the eye. PTX3 was also upregulated following *B. cereus* infection, and is known to be produced in response to TLR engagement [17]. PTX3 activates the classical complement pathway via C1q [18]. Therefore it could be hypothesized that PTX3 might function to activate an anti-bacterial response to *B. cereus* intraocular infection. However, it is currently unknown whether complement plays a role in *B. cereus* endophthalmitis. While complement is present in the eye [44], its absence did not alter the outcome in a mouse model of *S. aureus* endophthalmitis [45].

SOCS3 is a negative regulator of cytokine signaling induced by IL-6, IL-10, and INFγ (mediators of both the MYD88-dependent and MYD88-independent TLR pathways). SOCS3 functions to inhibit STAT3 phosphorylation and this negative regulatory function prevents excessive activation of proinflammatory genes [19]. The rapid and destructive inflammatory response observed following *B. cereus* infection suggests that SOCS3 may not adequately inhibit STAT3 phosphorylation in the cells in the retina which function as the initial responders. Wang et al. demonstrated that prolonged STAT3 activation occurs as a result of the IL-6 receptor associating with the epidermal growth factor receptor [19]. This complex is capable of STAT3 activation but is not inhibited by SOCS3. *The pathogen Mycobacterium tuberculosis* directly activates SOCS3 and therefore inhibits NF-κB/rel-mediated proinflammatory cytokine production [46], which functions to suppress the inflammatory response. It is currently unknown as to whether *B. cereus* is capable of directly activating SOCS3 in the retina, however the robust inflammatory response incited by *B. cereus* might override the inhibitory effects of SOCS3 activation.

LIF upregulation in response to *B. cereus* infection might serve to enhance the protection of the delicate, nonregenerative photoreceptors. LIF is an IL-6 regulated neurocytokine that is upregulated in Muller cells in response to retinal stress [47, 48]. Chucair-Elliott et al. demonstrated that LIF downregulates the expression of RPE65, which ultimately leads to a decrease in 11-cis-retinal, a chromophore that might be toxic in excessive amounts [49]. *B. cereus* infection may be a stressor that results in upregulation of LIF in order to protect this layer of cells in the retina. While it is unknown which cells are expressing LIF at increased levels following infection, given that Muller cells upregulate LIF in response to stress [47, 48], Muller cells might serve as at least one of the cell types that produce LIF in response to *B. cereus* infection.

Induction of PTGS2/COX-2 during infection is mediated by TLR4 and NFκB (nuclear factor kappa-light-chain-enhancer of activated B cells) [50]. This enzyme converts arachidonic acid to prostaglandin endoperoxide H2 (PGE_2_) and is expressed during inflammation. PGE_2_ has an immunomodulatory effect and serves to prevent the activation of neutrophils, which is an immune evasion strategy utilized by some bacterial pathogens. *Streptococcus pneumoniae* induces PGE_2_ production by human neutrophils and prevents activation [51]. In a model of *Pseudomonas* pneumonia, lack of PTGS2/COX-2 was proven to be beneficial and resulted in increased clearance of bacteria from the lungs [52]. The mechanism for this was linked to PGE_2_ inhibiting superoxide production by immune effectors and therefore hindering bacterial killing. PTGS2/COX-2 is usually an inflammation inducible enzyme not normally expressed in most tissues. However, PTGS2/COX-2 is constitutively expressed throughout both murine and human eyes [53], in the cornea, iris, ciliary body, and retina. Wang et al. suggested that PTGS2/COX-2 might play a protective role in eye tumorigenesis. Significant upregulation of PTGS2/COX-2 and PGE_2_ synthesis in the retina following *B. cereus* infection might serve to modulate the function of invading neutrophils and prevent activation.

The fact that 15 genes associated with TLR4 activation were upregulated 5-fold or greater in C57BL6/J mice compared to uninfected control eyes, but were not induced in TLR4^−/−^ suggests that *B. cereus* is capable of activation of TLR4. This implicates a component of *B. cereus* as a novel ligand for activating the TLR4 receptor. TLR4 typically mediates the inflammatory response to LPS in conjunction with MD2 (lymphocyte antigen 96), CD14, and MYD88 [54]. However, TLR4 has been shown to recognize additional exogenous and endogenous ligands, including respiratory syncytial virus, heat-shock proteins, fibronectin, fibrinogen, and hyaluronic acid [55–61]. Alternatively, *B. cereus* might instigate a response that results in the formation of an endogenous ligand for the TLR4 pathway. In the current study, we did not observe a transcriptional upregulation of any of the reported endogenous ligands. However, we cannot rule out the possibility that *B. cereus* infection might result in the posttranscriptional production or modification of an endogenous ligand.

The ability of *B. cereus* to activate both the TLR4 [8] and TLR2 [21] pathways might explain why *B. cereus* endophthalmitis results in an explosive inflammatory response that results in poor visual outcomes in affected patients. The early onset of TLR4-associated inflammation could play a key role in therapies designed to prevent further inflammation and damage to the sensitive and nonregenerative structures of the eye. This study identified retinal genes that were significantly upregulated early during *B. cereus* infection that might prove tractable as targets for intervention. However, inherent redundancies in these pathways and the potential for exacerbating inflammation might complicate the design of new therapeutics. Our study also presented key differences between the inflammatory mediators that are elicited by *B. cereus* and those by *S. aureus* [29] following intraocular infection. Endophthalmitis severity and outcome as result of infection with these two pathogens is starkly different, and the results of this study shed light on the differences in types and timing of inflammatory mediator production that contribute to the distinctive courses and outcomes. Given the limitations inherent to microarray analysis for assessing global gene changes, future studies confirming these results by proteomics and the analysis of the severity of *B. cereus* infections in mice specifically deficient in these pathways will be required. Future studies will evaluate the retinal and global ocular inflammatory responses over the course of *B. cereus* endophthalmitis to identify pathway-based anti-inflammatory targets, specifically, those that are regulated by TLR4, and to identify the *B. cereus* produced or induced ligand for TLR4.

## Conclusions

Our results have identified key proinflammatory and immunomodulatory mediators driven by TLR4 that may be important in regulating pro-inflammatory and protective pathways in the retina in response to *B. cereus* intraocular infection. These factors were upregulated early during infection and might serve as targets for new therapies. Our results also support the prospect that blocking the activation of TLR-based pathways might serve as alternative targets for Gram-positive endophthalmitis therapies in general.

## Additional files

Additional file 1: **Table S2.** Quantitative PCR primers. Sequences of the primers used for quantitative PCR analysis of mouse retinal gene expression. (DOCX 13 kb)

Additional file 2: **Table S1.** Microarray analysis of retinal gene expression following *B. cereus* infection. Retinal genes differentially expressed 2- to 4.9-fold 4 h postinfection with *B. cereus* ATCC14579. (DOCX 20 kb)

Additional file 3: **Figure S1**. Ingenuity Pathway analysis of 5-fold upregulated genes. Ingenuity Pathway Analysis of acute proinflammatory response and inflammatory cell recruitment genes upregulated 5-fold or greater following *B. cereus* ATCC14579 infection. (PDF 153 kb)

Additional file 4: **Figure S2.** Ingenuity Pathway analysis of 5-fold upregulated genes. Ingenuity Pathway Analysis of neutrophil recruitment and pathogen recognition genes upregulated 5-fold or greater following *B. cereus* ATCC14579 infection. (PDF 153 kb)

Additional file 5: **Figure S3.** Ingenuity Pathway analysis of 5-fold upregulated genes. Ingenuity Pathway Analysis of genes encoding regulators of the inflammatory response upregulated 5-fold or greater following *B. cereus* ATCC14579 infection. (PDF 155 kb)

## Abbreviations

IL-1β: Interleukin 1 beta
CCL2: Chemokine (C-C motif) ligand 2
CCL3: Chemokine (C-C motif) ligand 1
CFU: Colony-forming units
CH25H: Cholesterol 25-hydroxylase
COX-2 or PTGS2: Cyclooxgenase isoenzyme
C_T_: Threshold cycle
CXCL1: Chemokine (C-X-C motif) ligand 1
CXCL10: Chemokine (C-X-C motif) ligand 10
CXCL2: Chemokine (C-X-C motif) ligand 2
CYR61: Cysteine rich protein 61
EE: Endogenous endophthalmitis
EGR2: Early growth response 2 gene
ICAM1: Intercellular adhesion molecule 1
IFN-γ: Interferon gamma
IL-6: Interleukin 6
IP-10: Interferon gamma-induced protein 10
KC: Keratinocyte chemoattractant
LFA-1: Lymphocyte function-associated antigen 1
LIF: Leukemia inhibitory factor
MBP: Myelin basic protein
MCP1: Monocyte chemoattractant protein 1
MD2: Lymphocyte antigen 96
MIP1-α: Macrophage inflammatory protein 1-alpha
MIP2-α: Macrophage inflammatory protein 2-alpha
MOBP: Myelin-associated oligodendrocytic basic protein
NFκB: Nuclear factor kappa-light-chain-enhancer of activated B cells
PGE_2_: Prostaglandin endoperoxide H2
PLP1: Proteolipid protein 1
PMN: Polymorphonuclear leukocytes
POE: Post-operative endophthalmitis
PTE: Post-traumatic endophthalmitis
PTX3: Pentraxin 3
qPCR: Quantitative polymerase chain reaction
RANTES: Regulated on activation, normal T cell expressed and secreted
SOCS3: Suppressor of cytokine signaling 3
STAT3: Signal transducer and activator of transcription 3
STEAP4: Six transmembrane epithelial antigen of prostate, family member 4
TLR: Toll-like receptors
TNF-α: Tumor necrosis factor alpha

## References

[1] Callegan MC, Gilmore MS, Gregory M, Ramadan RT, Wiskur BJ, Moyer AL (2007). Bacterial endophthalmitis: therapeutic challenges and host–pathogen interactions. Prog Retin Eye Res.

[2] Callegan MC, Kane ST, Cochran DC, Gilmore MS (2002). Molecular mechanisms of *Bacillus* endophthalmitis pathogenesis. DNA Cell Biol.

[3] Coburn PS, Callegan MC. Endophthalmitis. In: Rumelt S, editor. Advances in ophthalmology. InTech. 10.5772/29130. Available from: https://www.intechopen.com/books/advances-in-ophthalmology/endophthalmitis.

[4] Durand ML (2013). Endophthalmitis. Clin Microbiol Infect.

[5] Callegan MC, Kane ST, Cochran DC, Gilmore MS, Gominet M, Lereclus D (2003). Relationship of *plcR*-regulated factors to *Bacillus* endophthalmitis virulence. Infect Immun.

[6] Ramadan RT, Ramirez R, Novosad BD, Callegan MC (2006). Acute inflammation and loss of retinal architecture and function during experimental *Bacillus* endophthalmitis. Curr Eye Res.

[7] Ramadan RT, Moyer AL, Callegan MC (2008). A role for tumor necrosis factor-alpha in experimental *Bacillus cereus* endophthalmitis pathogenesis. Invest Ophthalmol Vis Sci.

[8] Parkunan SM, Randall CB, Coburn PS, Astley RA, Staats RL, Callegan MC (2015). Unexpected roles for toll-like receptor 4 and TRIF in intraocular infection with gram-positive bacteria. Infect Immun.

[9] Parkunan SM, Randall CB, Astley RA, Furtado GC, Lira SA, Callegan MC (2016). CXCL1, but not IL-6, significantly impacts intraocular inflammation during infection. J Leukoc Biol.

[10] David DB, Kirkby GR, Noble BA (1994). *Bacillus cereus* endophthalmitis. Br J Ophthalmol.

[11] Akira S, Takeda K (2004). Toll-like receptor signalling. Nature Rev Immuno.

[12] Kawai T, Akira S (2010). The role of pattern-recognition receptors in innate immunity: update on toll-like receptors. Nat Immun.

[13] Hoshino K, Takeuchi O, Kawai T, Sanjo H, Ogawa T, Takeda Y, Takeda K, Akira S (1999). Cutting edge: toll-like receptor 4 (TLR4)-deficient mice are hyporesponsive to lipopolysaccharide: evidence for TLR4 as the Lps gene product. J Immunol.

[14] Rothlein R, Dustin ML, Marlin SD, Springer TA (1986). A human intercellular adhesion molecule (ICAM-1) distinct from LFA-1. J Immunol.

[15] Dustin ML, Rothlein R, Bhan AK, Dinarello CA, Springer TA (1986). Induction by IL 1 and interferon-gamma: tissue distribution, biochemistry, and function of a natural adherence molecule (ICAM-1). J Immunol.

[16] Wiedmaier N, Müller S, Köberle M, Manncke B, Krejci J, Autenrieth IB, Bohn E (2008). Bacteria induce CTGF and CYR61 expression in epithelial cells in a lysophosphatidic acid receptor-dependent manner. Int J Med Microbiol.

[17] Bottazzi B, Garlanda C, Cotena A (2009). The long pentraxin PTX3 as a prototypic humoral pattern recognition receptor: interplay with cellular innate immunity. Immuno Rev.

[18] Nauta AJ, Bottazzi B, Mantovani A, Salvatori G, Kishore U, Schwaeble WJ, Gingras AR, Tzima S, Vivanco F, Egido J, Tijsma O, Hack EC, Daha MR, Roos A (2003). Biochemical and functional characterization of the interaction between pentraxin 3 and C1q. Eur J Immunol.

[19] Wang Y, van Boxel-Dezaire AHH, Cheon H, Yang J, Stark GR (2013). STAT3 activation in response to IL-6 is prolonged by the binding of IL-6 receptor to EGF receptor. Proc Natl Acad Sci.

[20] Scarl RT, Lawrence CM, Gordon HM, Nunemaker CS (2017). STEAP4: its emerging role in metabolism and homeostasis of cellular iron and copper. J Endocrinol.

[21] Novosad BD, Astley RA, Callegan MC (2011). Role of toll-like receptor (TLR) 2 in experimental *Bacillus cereus* endophthalmitis. PLoS One.

[22] Talreja D, Singh PK, Kumar A (2015). In vivo role of TLR2 and MyD88 signaling in eliciting innate immune responses in staphylococcal endophthalmitis. Invest Ophthalmol Vis Sci.

[23] Hunt JJ, Astley R, Wheatley N, Wang JT, Callegan MC (2014). TLR4 contributes to the host response to *Klebsiella* intraocular infection. Curr Eye Res.

[24] Wolpe SD, Sherry B, Juers D, Davatelis G, Yurt RW, Cerami A (1989). Identification and characterization of macrophage inflammatory protein 2. Proc Nat Acad Sci.

[25] Iida N, Grotendorst GR (1990). Cloning and sequencing of a new gro transcript from activated human monocytes: expression in leukocytes and wound tissue. Mol Cell Biol.

[26] Pelus LM, Fukuda S (2006). Peripheral blood stem cell mobilization: the CXCR2 ligand GRObeta rapidly mobilizes hematopoietic stem cells with enhanced engraftment properties. Exp Hematol.

[27] Dufour JH, Dziejman M, Liu MT, Leung JH, Lane TE, Luster AD (2002). IFN-gamma-inducible protein 10 (IP-10; CXCL10)-deficient mice reveal a role for IP-10 in effector T cell generation and trafficking. J Immunol.

[28] Luster AD, Unkeless JC, Ravetch JV (1985). Gamma-interferon transcriptionally regulates an early-response gene containing homology to platelet proteins. Nature.

[29] Rajamani D, Singh PK, Rottmann BG, Singh N, Bhasin MK, Kumar A (2016). Temporal retinal transcriptome and systems biology analysis identifies key pathways and hub genes in *Staphylococcus aureus* endophthalmitis. Sci Rep.

[30] Tilg H, Trehu E, Atkins MB, Dinarello CA, Mier JW (1994). Interleukin-6 (IL-6) as an anti-inflammatory cytokine: induction of circulating IL-1 receptor antagonist and soluble tumor necrosis factor receptor p55. Blood.

[31] Elner VM, Scales W, Elner SG, Danforth J, Kunkel SL, Strieter RM (1992). Interleukin-6 (IL-6) gene expression and secretion by cytokine-stimulated human retinal pigment epithelial cells. Exp Eye Res.

[32] Planck SR, Dang TT, Graves D, Tara D, Ansel JC, Rosenbaum JT (1992). Retinal pigment epithelial cells secrete interleukin-6 in response to interleukin-1. Invest Ophthalmol Vis Sci.

[33] Leibinger M, Müller A, Gobrecht P, Diekmann H, Andreadaki A, Fischer D (2013). Interleukin-6 contributes to CNS axon regeneration upon inflammatory stimulation. Cell Death Dis.

[34] Sappington RM, Chan M, Calkins DJ (2006). Interleukin-6 protects retinal ganglion cells from pressure-induced death. Invest Ophthalmol Vis Sci.

[35] Carr MW, Roth SJ, Luther E, Rose SS, Springer TA (1994). Monocyte chemoattractant protein 1 acts as a T-lymphocyte chemoattractant. Proc Natl Acad Sci U S A.

[36] Xu LL, Warren MK, Rose WL, Gong W, Wang JM (1996). Human recombinant monocyte chemotactic protein and other C-C chemokines bind and induce directional migration of dendritic cells in vitro. J Leukoc Biol.

[37] Xue ML, Thakur A, Cole N, Lloyd A, Stapleton F, Wakefield D, Willcox MD (2007). A critical role for CCL2 and CCL3 chemokines in the regulation of polymorphonuclear neutrophils recruitment during corneal infection in mice. Immunol Cell Biol.

[38] Giese MJ, Shum DC, Rayner SA, Mondino BJ, Berliner JA (2000). Adhesion molecule expression in a rat model of *Staphylococcus aureus* endophthalmitis. Invest Ophthalmol Vis Sci.

[39] Roebuck KA, Finnegan A (1999). Regulation of intercellular adhesion molecule-1 (CD54) gene expression. J Leukoc Biol.

[40] Cho RL, Yang CC, Lee IT, Lin CC, Chi PL, Hsiao LD, Yang CM (2016). Lipopolysaccharide induces ICAM-1 expression via a c-Src/NADPH oxidase/ROS-dependent NF-κB pathway in human pulmonary alveolar epithelial cells. Am J Physiol Lung Cell Mol Physiol.

[41] Yang L, Froio RM, Sciuto TE, Dvorak AM, Alon R, Luscinskas FW (2005). ICAM-1 regulates neutrophil adhesion and transcellular migration of TNF-α-activated vascular endothelium under flow. Blood.

[42] Blaber R, Stylianou E, Clayton A, Steadman R (2003). Selective regulation of ICAM-1 and RANTES gene expression after ICAM-1 ligation on human renal fibroblasts. J Am Soc Nephrol.

[43] Clayton A, Evans RA, Pettit E, Hallett M, Williams JD, Steadman R (1998). Cellular activation through the ligation of intercellular adhesion molecule-1. J Cell Sci.

[44] Sohn JH, Kaplan HJ, Suk HJ, Bora PS, Bora NS (2000). Chronic low level complement activation within the eye is controlled by intraocular complement regulatory proteins. Invest Ophthalmol Vis Sci.

[45] Engelbert M, Gilmore MS (2005). Fas ligand but not complement is critical for control of experimental *Staphylococcus aureus* Endophthalmitis. Invest Ophthalmol Vis Sci.

[46] Nair S, Pandey AD, Mukhopadhyay S (2011). The PPE18 protein of *Mycobacterium tuberculosis* inhibits NF-κB/rel-mediated proinflammatory cytokine production by upregulating and phosphorylating suppressor of cytokine signaling 3 protein. J Immunol.

[47] Joly S, Lange C, Thiersch M, Samardzija M, Grimm C (2008). Leukemia inhibitory factor extends the lifespan of injured photoreceptors *in vivo*. J Neurosci.

[48] Bürgi S, Samardzija M, Grimm C (2009). Endogenous leukemia inhibitory factor protects photoreceptor cells against light-induced degeneration. Mol Vis.

[49] Chucair-Elliott AJ, Elliott MH, Wang J, Moiseyev GP, Ma JX, Politi LE, Rotstein NP, Akira S, Uematsu S, Ash JD (2012). Leukemia inhibitory factor coordinates the down-regulation of the visual cycle in the retina and retinal-pigmented epithelium. J Biol Chem.

[50] Rhee SH, Hwang D (2000). Murine TOLL-like receptor 4 confers lipopolysaccharide responsiveness as determined by activation of NF kappa B and expression of the inducible cyclooxygenase. J Biol Chem.

[51] Cockeran R, Steel HC, Mitchell TJ, Feldman C, Anderson R (2001). Pneumolysin potentiates production of prostaglandin E(2) and leukotriene B(4) by human neutrophils. Infect Immun.

[52] Sadikot RT, Zeng H, Azim AC, Joo M, Dey SK, Breyer RM, Peebles RS, Blackwell TS, Christman JW (2007). Bacterial clearance of *Pseudomonas aeruginosa* is enhanced by the inhibition of COX-2. Eur J Immunol.

[53] Wang J, Wu Y, Heegaard S, Kolko M (2011). Cyclooxygenase-2 expression in the normal human eye and its expression pattern in selected eye tumours. Acta Ophthalmol.

[54] Zhang G, Ghosh S (2001). Toll-like receptor-mediated NF-kappaB activation: a phylogenetically conserved paradigm in innate immunity. J Clin Invest.

[55] Smiley ST, King JA, Hancock WW (2001). Fibrinogen stimulates macrophage chemokine secretion through toll-like receptor 4. J Immunol.

[56] Lee JY, Sohn KH, Rhee SH, Hwang D (2001). Saturated fatty acids, but not unsaturated fatty acids, induce the expression of cyclooxygenase-2 mediated through toll-like receptor 4. J Biol Chem.

[57] Termeer C, Benedix F, Sleeman J, Fieber C, Voith U, Ahrens T, Miyake K, Freudenberg M, Galanos C, Simon JC (2002). Oligosaccharides of Hyaluronan activate dendritic cells via toll-like receptor 4. J Exp Med.

[58] Ohashi K, Burkart V, Flohe S, Kolb H (2000). Cutting edge: heat shock protein 60 is a putative endogenous ligand of the toll-like receptor-4 complex. J Immunol.

[59] Kiechl S, Lorenz E, Reindl M, Wiedermann CJ, Oberhollenzer F, Bonora E, Willeit J, Schwartz DA (2002). Toll-like receptor 4 polymorphisms and atherogenesis. N Engl J Med.

[60] Haeberle HA, Takizawa R, Casola A, Brasier AR, Dieterich HJ, Van Rooijen N, Gatalica Z, Garofalo RP (2002). Respiratory syncytial virus-induced activation of nuclear factor-kappaB in the lung involves alveolar macrophages and toll-like receptor 4-dependent pathways. J Infect Dis.

[61] Okamura Y, Watari M, Jerud ES, Young DW, Ishizaka ST, Rose J, Chow JC, Strauss JF (2001). The extra domain a of fibronectin activates toll-like receptor 4. J Biol Chem.

