# Supplementary material for: TLR4 modulates inflammatory gene targets in the retina during Bacillus cereus endophthalmitis
Source: BMC Ophthalmol. 2018 Apr 16;18:96. doi: 10.1186/s12886-018-0764-8 (PMC5902844; doi:10.1186/s12886-018-0764-8)
Supplement: Supplementary file 1 — Table S2. Quantitative PCR primers. Sequences of the primers used for quantitative PCR analysis of mouse retinal gene expression. (DOCX 13 kb) [file 12886_2018_764_MOESM1_ESM.docx]

Table S2 – qPCR Primers

| Gene name | Primer sequences (5’-3’) |
| --- | --- |
| CCL2 | Sense primer: 5’- CAT CCA CGT GTT GGC TCA - 3’  Anti-sense primer: 5’- AAC TAC AGC TTC TTT GGG ACA - 3’ |
| IL6 | Sense primer: 5’ - GAT ACC ACT CCC AAC AGA CC - 3’  Anti-sense primer: 5’ - CAA GTG CAT CAT CGT TGT TCA - 3’ |
| CXCL2 | Sense primer: 5’ - CAG AAG TCA TAG CCA CTC TCA AG - 3’  Anti-sense primer: 5’ - CTT TCC AGG TCA GTT AGC CTT - 3’ |
| CCL3 | Sense primer: 5’ - CCT TGC TGT TCT TCT CTG TAC C - 3’  Anti-sense primer: 5’ - CGA TGA ATT GGC GTG GAA TC - 3’ |
| PTX3 | Sense primer: 5’ - CGA CAG TGC CTG GCC TA - 3’  Anti-sense primer: 5’ - TTC GAA CGC ATT GGG AAG A - 3’ |
| CXCL1 | Sense primer: 5’ - CCA AAC CGA AGT CAT AGC CA - 3’  Anti-sense primer: 5’ - GTG CCA TCA GAG CAG TCT - 3’ |
| LIF | Sense primer: 5’ - GCA ACC TCA TGA ACC AGA TCA - 3’  Anti-sense primer: 5’ - GCA CAT AGC TTT TCC ACG TTG - 3’ |
| CXCL10 | Sense primer: 5’ - ATT TTC TGC CTC ATC CTG CT - 3’  Anti-sense primer: 5’ - TGA TTT CAA GCT TCC CTA TGG C - 3’ |
| ICAM1 | Sense primer: 5’ - CGC TGC TAC CTG CAC TT - 3’  Anti-sense primer: 5’ - GAT GGA TAC CTG AGC ATC ACC - 3’ |
| SOCS3 | Sense primer: 5’ - GAG ATT TCG CTT CGG GAC TA - 3’  Anti-sense primer: 5’ - GGA AAC TTG CTG TGG GTG A - 3’ |
| PTGS2 | Sense primer: 5’ - CAC CTC TGC GAT GCT CTT C - 3’  Anti-sense primer: 5’ - ATC CTG TGC TCA TAC ATT CCC - 3’ |
