# Supplementary material for: TLR4 modulates inflammatory gene targets in the retina during Bacillus cereus endophthalmitis
Source: BMC Ophthalmol. 2018 Apr 16;18:96. doi: 10.1186/s12886-018-0764-8 (PMC5902844; doi:10.1186/s12886-018-0764-8)
Supplement: Supplementary file 2 — Table S1. Microarray analysis of retinal gene expression following B. cereus infection. Retinal genes differentially expressed 2- to 4.9-fold 4 h postinfection with B. cereus ATCC14579. (DOCX 20 kb) [file 12886_2018_764_MOESM2_ESM.docx]

S1 Table. Retinal genes differentially expressed 2- to 4.9-fold 4 hours postinfection with *B. cereus* ATCC14579. NC = No Change; NS = Not significant

| Gene  Symbol | Gene Title | RefSeq Transcript ID | Fold-Change  (C57BL6J infected  versus uninfected) | p-value | Fold-Change  (TLR4^-/-^ infected  versus uninfected) | p-value |
| --- | --- | --- | --- | --- | --- | --- |
| Zfp36 | zinc finger protein 36 | NM_011756 | 5 | 0.0052 | NC | NS |
| Cebpd | CCAAT/enhancer binding protein delta | NM_007679 | 4 | 0.0038 | NC | NS |
| Maff | v-maf musculoaponeurotic fibrosarcoma oncogene family, protein F | NM_010755 | 4 | 0.0173 | NC | NS |
| Lcn2 | lipocalin 2 | NM_008491 | 4 | 0.0018 | NC | NS |
| Egr3 | early growth response 3 | NM_018781 | 4 | 0.0152 | NC | NS |
| Tnfaip2 | tumor necrosis factor, alpha-induced protein 2 | NM_009396 | 4 | 0.0147 | NC | NS |
| Serpine1 | serine (or cysteine) peptidase inhibitor | NM_008871 | 4 | 0.0095 | NC | NS |
| Fos | FBJ osteosarcoma oncogene | NM_010234 | 4 | 0.0054 | NC | NS |
| Adamts1 | a disintegrin-like and metallopeptidase with thrombospondin type 1 motif | NM_009621 | 4 | 0.0093 | NC | NS |
| Irf1 | interferon regulatory factor 1 | NM_008390 | 4 | 0.0013 | NC | NS |
| Mbd1 | methyl-CpG binding domain protein 1 | NM_013594 | 4 | 0.0017 | NC | NS |
| F3 | coagulation factor III | NM_010171 | 3 | 0.0077 | NC | NS |
| Cebpb | CCAAT/enhancer binding protein beta | NM_009883 | 3 | 0.0019 | NC | NS |
| Junb | Jun-B oncogene | NM_008416 | 3 | 0.0059 | NC | NS |
| Tlr2 | toll-like receptor 2 | NM_011905 | 3 | 0.0131 | NC | NS |
| Krt6a | keratin 6A | NM_008476 | 3 | 0.0102 | NC | NS |
| Mt1 | metallothionein 1 | NM_013602 | 3 | 0.0073 | NC | NS |
| Sox9 | SRY-box containing gene 9 | NM_011448 | 3 | 0.0183 | NC | NS |
| Tnfaip6 | tumor necrosis factor alpha induced protein 6 | NM_009398 | 3 | 0.0119 | NC | NS |
| Phlda1 | pleckstrin homology-like domain, family A | NM_009344 | 3 | 0.0231 | NC | NS |
| Nfkbiz | nuclear factor of kappa light polypeptide gene enhancer in B cells inhibitor, zeta | NM_030612 | 3 | 0.0004 | NC | NS |
| Ifi202b | interferon activated gene 202B | NM_008327 | 3 | 0.0490 | NC | NS |
| Dsp | desmoplakin | NM_023842 | 3 | 0.0372 | NC | NS |
| Osmr | oncostatin M receptor | NM_011019 | 3 | 0.0318 | NC | NS |
| Crispld2 | cysteine-rich secretory protein LCCL domain containing 2 | NM_030209 | 3 | 0.0074 | NC | NS |
| Calml3 | calmodulin-like 3 | NM_027416 | 3 | 0.0369 | NC | NS |
| Ccl7 | chemokine (C-C motif) ligand 7 | NM_013654 | 3 | 0.0432 | NC | NS |
| Rab20 | RAB20, member RAS oncogene family | NM_011227 | 3 | 0.0216 | NC | NS |
| A130040M12Rik | RIKEN cDNA A130040M12 gene | NR_002860 | 3 | 0.0241 | NC | NS |
| BC100530 | cDNA sequence BC100530 | NM_001082546 | 3 | 0.0165 | NC | NS |
| 1810011O10Rik | RIKEN cDNA 1810011O10 gene | NM_026931 | 3 | 0.0081 | NC | NS |
| Cldn11 | claudin 11 | NM_008770 | NC | NS | 3 | 0.0224 |
| Ugt8a | UDP galactosyltransferase 8A | NM_011674 | NC | NS | 3 | 0.0430 |
| Ermn | ermin, ERM-like protein | NM_029972 | NC | NS | 3 | 0.0221 |
| Gfap | glial fibrillary acidic protein | NM_010277 | NC | NS | 3 | 0.0084 |
| Gadd45b | growth arrest and DNA-damage-inducible 45 beta | NM_008655 | 2 | 0.0229 | NC | NS |
| Egr1 | early growth response 1 | NM_007913 | 2 | 0.0151 | NC | NS |
| Ets1 | E26 avian leukemia oncogene 1 | NM_011808 | 2 | 0.0090 | NC | NS |
| Bag3 | BCL2-associated athanogene 3 | NM_013863 | 2 | 0.0461 | NC | NS |
| Gadd45b | growth arrest and DNA-damage-inducible 45 beta | NM_008655 | 2 | 0.0115 | NC | NS |
| Zfp36l1 | zinc finger protein 36, C3H type-like 1 | NM_007564 | 2 | 0.0083 | NC | NS |
| Mt2 | metallothionein 2 | NM_008630 | 2 | 0.0154 | NC | NS |
| Ccl12 | chemokine (C-C motif) ligand 12 | NM_011331 | 2 | 0.0074 | NC | NS |
| Clcf1 | cardiotrophin-like cytokine factor 1 | NM_019952 | 2 | 0.0496 | NC | NS |
| Vcam1 | vascular cell adhesion molecule 1 | NM_011693 | 2 | 0.0029 | NC | NS |
| Arc | activity regulated cytoskeletal-associated protein | NM_018790 | 2 | 0.0151 | NC | NS |
| Ier2 | immediate early response 2 | NM_010499 | 2 | 0.0215 | NC | NS |
| Gcnt2 | glucosaminyl (N-acetyl) transferase 2, I-branching enzyme | NM_008105 | 2 | 0.0460 | NC | NS |
| Csrnp1 | cysteine-serine-rich nuclear protein 1 | NM_153287 | 2 | 0.0011 | NC | NS |
| Isg15 | ISG15 ubiquitin-like modifier | NM_015783 | 2 | 0.0419 | NC | NS |
| Id2 | inhibitor of DNA binding 2 | NM_010496 | 2 | 0.0234 | NC | NS |
| Il1a | interleukin 1 alpha | NM_010554 | 2 | 0.0072 | NC | NS |
| Ccl4 | chemokine (C-C motif) ligand 4 | NM_013652 | 2 | 0.0121 | NC | NS |
| 2010002N04Rik | RIKEN cDNA 2010002N04 gene | NM_134133 | 2 | 0.0302 | NC | NS |
| C730049O14Rik | RIKEN cDNA C730049O14 gene | --- | 2 | 0.0237 | NC | NS |
| Rnd1 | Rho family GTPase 1 | NM_172612 | 2 | 0.0482 | NC | NS |
| Csf3 | colony stimulating factor 3 (granulocyte) | NM_009971 | 2 | 0.0068 | NC | NS |
| Fas | Fas (TNF receptor superfamily member 6) | NM_007987 | 2 | 0.0059 | NC | NS |
| Ier3 | immediate early response 3 | NM_133662 | 2 | 0.0057 | NC | NS |
| Mt1 | metallothionein 1 | NM_013602 | 2 | 0.0129 | NC | NS |
| Fosl2 | fos-like antigen 2 | NM_008037 | 2 | 0.0226 | NC | NS |
| Cd14 | CD14 antigen | NM_009841 | 2 | 0.0079 | NC | NS |
| Relb | avian reticuloendotheliosis viral (v-rel) oncogene related B | NM_009046 | 2 | 0.0036 | NC | NS |
| Klf6 | Kruppel-like factor 6 | NM_011803 | 2 | 0.0278 | NC | NS |
| Apold1 | apolipoprotein L domain containing 1 | NM_001109914 | 2 | 0.00003 | NC | NS |
| Apod | apolipoprotein D | NM_007470 | NC | NS | 2 | 0.0168 |
| Foxg1 | forkhead box G1 | NM_008241 | NC | NS | -2 | 0.0243 |
