# Supplementary material for: TLR4 modulates inflammatory gene targets in the retina during Bacillus cereus endophthalmitis
Source: BMC Ophthalmol. 2018 Apr 16;18:96. doi: 10.1186/s12886-018-0764-8 (PMC5902844; doi:10.1186/s12886-018-0764-8)

S1 Fig. Ingenuity Pathway Analysis of acute proinflammatory response and inflammatory cell recruitment genes upregulated 5-fold or greater following *B. cereus* ATCC14579 infection.

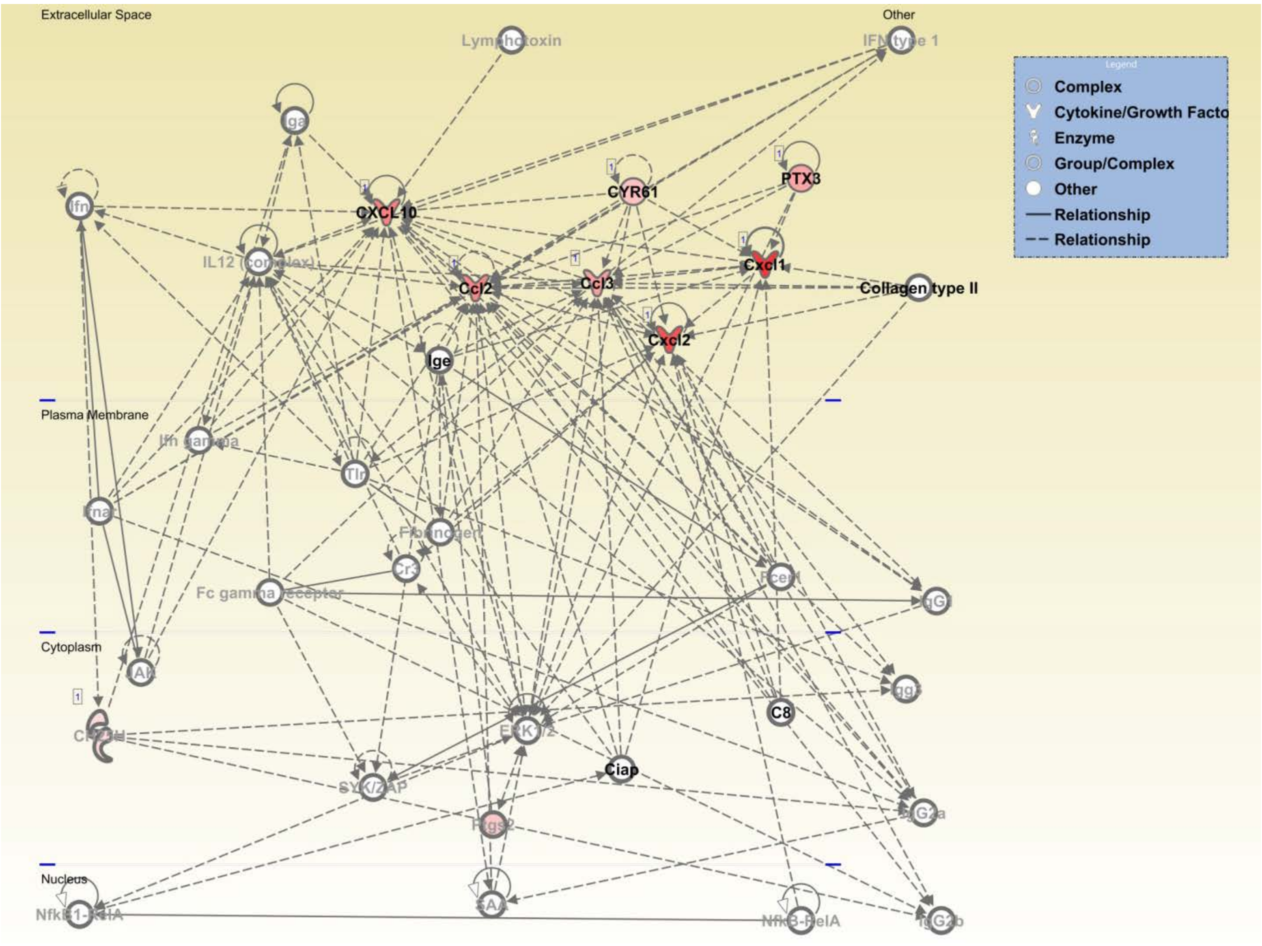

Supplement: Supplementary file 3 — Figure S1. Ingenuity Pathway analysis of 5-fold upregulated genes. Ingenuity Pathway Analysis of acute proinflammatory response and inflammatory cell recruitment genes upregulated 5-fold or greater following B. cereus ATCC14579 infection. (PDF 153 kb) [file 12886_2018_764_MOESM3_ESM.pdf]
