# Supplementary material for: TLR4 modulates inflammatory gene targets in the retina during Bacillus cereus endophthalmitis
Source: BMC Ophthalmol. 2018 Apr 16;18:96. doi: 10.1186/s12886-018-0764-8 (PMC5902844; doi:10.1186/s12886-018-0764-8)

S3 Fig. Ingenuity Pathway Analysis of genes encoding regulators of the inflammatory response upregulated 5-fold or greater following *B. cereus* ATCC14579 infection.

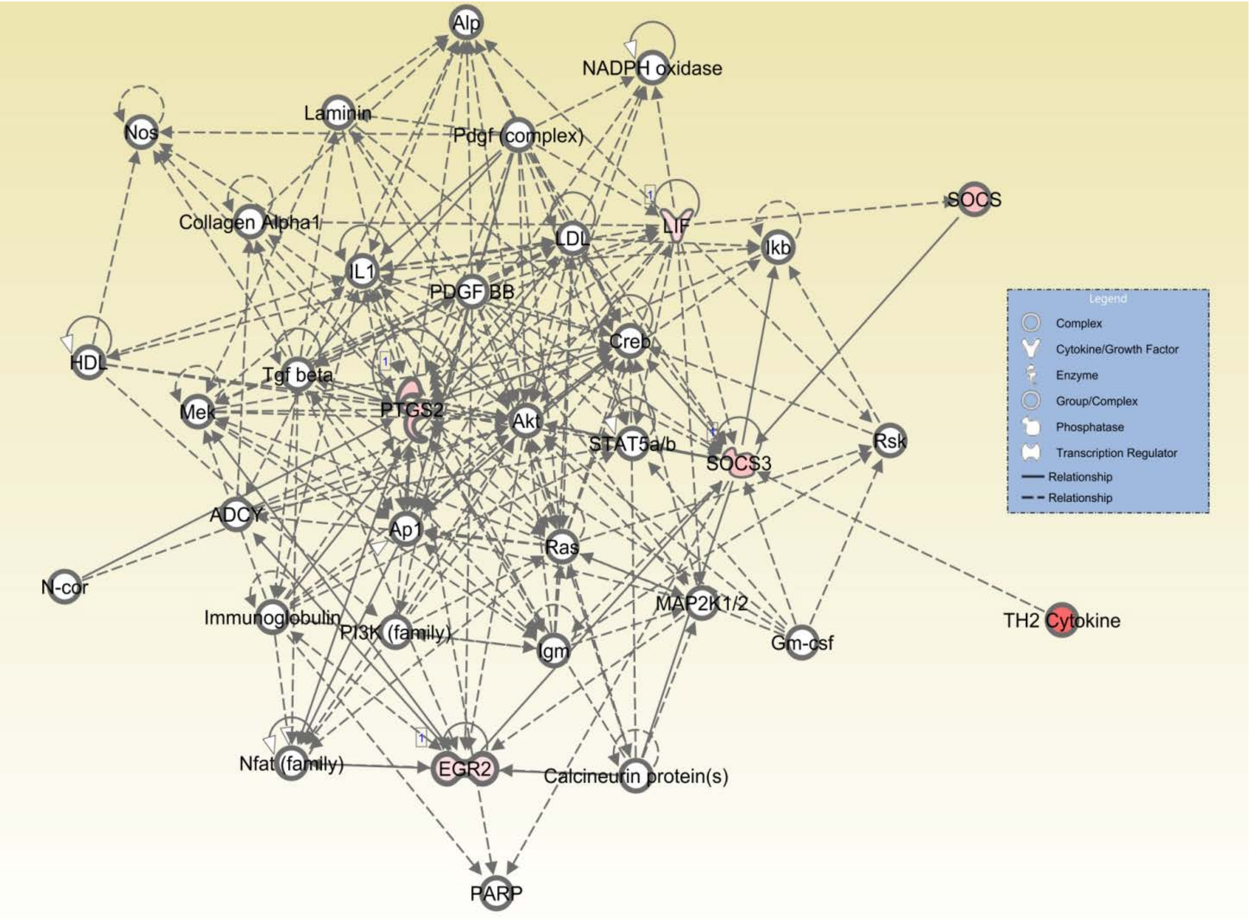

Supplement: Supplementary file 5 — Figure S3. Ingenuity Pathway analysis of 5-fold upregulated genes. Ingenuity Pathway Analysis of genes encoding regulators of the inflammatory response upregulated 5-fold or greater following B. cereus ATCC14579 infection. (PDF 155 kb) [file 12886_2018_764_MOESM5_ESM.pdf]
